# MAFF alleviates hepatic ischemia–reperfusion injury by regulating the CLCF1/STAT3 signaling pathway

**DOI:** 10.1186/s11658-025-00721-x

**Published:** 2025-04-01

**Authors:** Dengliang Lei, Yihua Wang, Shanshan Li, Song Xiang, Yunhai Luo, Ping Yan, Fang Luo, Zuotian Huang, ZhongJun Wu

**Affiliations:** 1 Department of Hepatobiliary Surgery, The First Affiliated Hospital of Chongqing Medical University, Chongqing, China; 2 Department of Hepatobiliary Pancreatic Tumor Center, Chongqing University Cancer Hospital, Chongqing, China; 3 Department of Hepatobiliary Surgery, Affiliated Hospital of Guizhou Medical University, Guizhou, China

**Keywords:** MAFF, Hepatic ischemia–reperfusion, BACH1, CLCF1, STAT3

## Abstract

**Background:**

Although hepatic ischemia–reperfusion injury (IRI) frequently occurs during liver resection and transplantation, the underlying mechanisms remain incompletely understood. Through high-throughput sequencing, we found that v-maf musculoaponeurotic fibrosarcoma oncogene homolog F (MAFF) expression was significantly increased after hepatic IRI. The specific role of MAFF, a basic leucine zipper (bZIP) transcription factor, in hepatic IRI is unknown. In the present study, we aimed to explore the effect of MAFF on hepatic IRI injury.

**Approach and results:**

Adenovirus vectors carrying the MAFF gene were administered to mice to explore the potential significance of MAFF. After ischemia–reperfusion, MAFF expression was significantly upregulated, suggesting a potential association between MAFF expression and hepatocyte apoptosis. A reduction in MAFF expression was demonstrated to worsen hepatic impairment and enhance the expression of proinflammatory cytokines in mice following ischemia–reperfusion. Conversely, MAFF overexpression had the opposite effect. Mechanistically, the combination of CUT&Tag and RNA sequencing technologies identified cardiotrophic factor-like cytokine 1 (CLCF1) as a direct transcriptional target for MAFF and BTB and CNC homology 1 (BACH1) heterodimers. This interaction subsequently triggers signal transducer and activator of transcription 3 (STAT3) signaling.

**Conclusions:**

MAFF alleviates hepatic ischemia–reperfusion injury by reducing hepatocyte apoptosis and the inflammatory response through the activation of the CLCF1/STAT3 signaling pathway, offering valuable insights into the impact of MAFF on liver protection and potential therapeutic targets for liver treatment.

**Graphical abstract:**

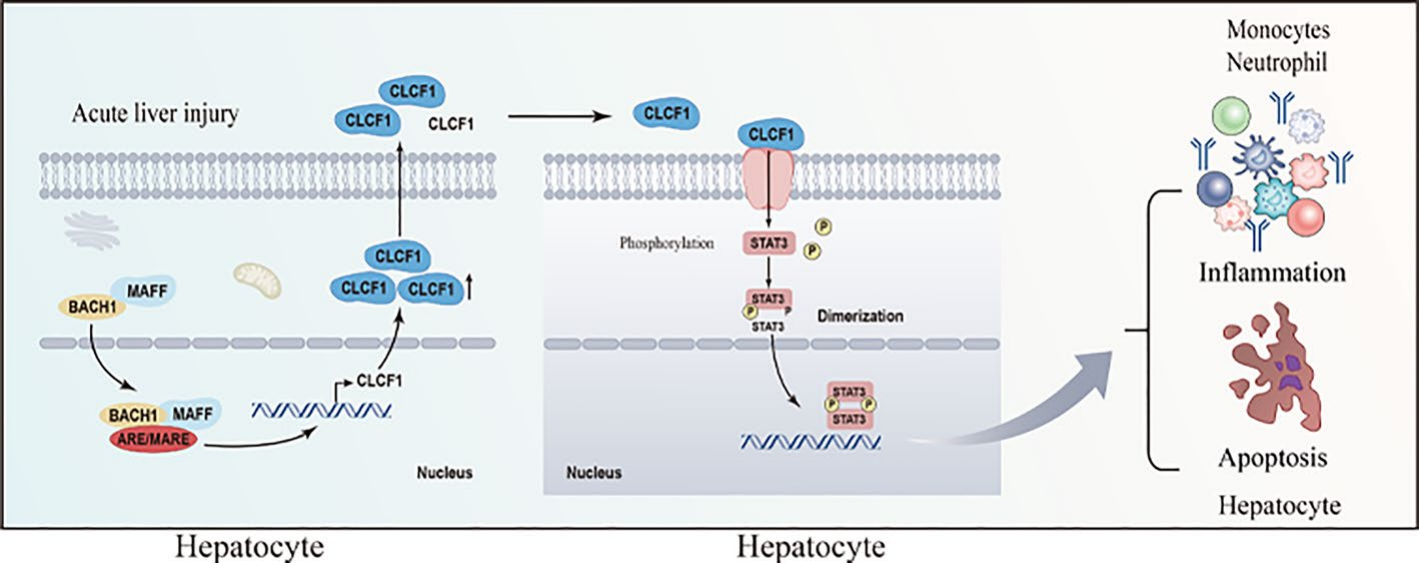

**Supplementary Information:**

The online version contains supplementary material available at 10.1186/s11658-025-00721-x.

## Introduction

Hepatic ischemia–reperfusion injury (IRI) is a significant contributor to liver damage following surgical interventions [1]. IRI can result in graft failure, tissue destruction, and, potentially, liver failure [2, 3]. Therefore, it is crucial to investigate the mechanisms underlying IRI to develop novel therapeutic strategies to address these clinical challenges.

The v-maf musculoaponeurotic fibrosarcoma oncogene homolog (MAF) family proteins were initially discovered as viral oncogenes in chickens [4]. These proteins are basic leucine zipper (bZIP) transcription factors that can be classified into two categories: large MAFs and small MAFs [4]. In contrast to their larger counterparts, such as MAFA (L-MAF), MAFB, c-MAF (MAF), and neura retinal (NRL), small MAF proteins, namely, MAFF, MAFK, and MAFG, do not possess transactivation domains [4]. Instead, they primarily form homodimers or heterodimers with members of the CNC family (NF-E2, NRF1, NRF2, and NRF3) and BACH family proteins (BACH1 and BACH2) [5–7]. The binding of tiny MAF proteins to the palindromic MAF recognition element (MARE) (5′-TGCTGACGTCAGCA-3′) or the antioxidant response element (ARE), which contains the core sequence 5′-R (A/G) T (C/G) A (C/T/G) NNNGC-3′, is necessary for CNC and BACH family members [8, 9]. Given that the MAF family is closely associated with oxidative stress, we conclude that members of the MAFF family may play a role in liver ischemia‒reperfusion injury.

Cardiotrophic factor-like cytokine 1 (CLCF1) is recognized for its neurotrophic properties and its ability to stimulate B cell activity [10, 11]. Impaired CLCF1 function results in cold-induced sweating syndrome, a pathological condition characterized by respiratory and neurological developmental abnormalities [12]. CLCF1 levels in cancer-associated fibroblasts play a role in promoting tumor cell stemness and facilitating the infiltration and polarization of tumor-associated neutrophils in hepatocellular carcinoma [13]. In lung cancer, CLCF1 acts as a protumorigenic factor in cancer-associated fibroblasts [14]. A recent study demonstrated that CLCF1, an interleukin-6 (IL-6) family cytokine involved in neurodevelopment, regulates the tumor immune milieu and has potential as a prognostic marker and therapeutic target in patients with glioma associated with phosphatase and tensin homolog (PTEN) mutations [15]. Although CLCF1 is closely associated with inflammatory infiltration, its role in hepatic ischemia–perfusion remains unclear.

Our study elucidated the significant role of MAFF in protecting against liver IRI. Our findings revealed that the combination of MAFF and BACH1 effectively reduces hepatic IRI by inducing CLCF1 transcription and activating the STAT3 pathway.

## Materials and methods

### Animals

All experimental procedures were approved by the Institutional Animal Care Committee of Chongqing Medical University and were conducted in accordance with the Basel Declaration (approval no. IACUC-CQMU2023-10056; date, 25 October 2023). The mice used in this study belonged to the C57BL/6 strain and were housed within a facility that maintained specific pathogen-free conditions. The Institutional Animal Care and Use Committee of Chongqing Medical University granted approval for all experimental procedures and methodologies.

### Animal model

The mice were subjected to a fasting regimen and anesthetized using a ketamine/xylazine combination. After midline laparotomy surgery, the mice were given heparin, and a nontraumatic clip was used to induce obstruction of blood flow to the left lateral/median lobes (70%) of the liver. After 90 min of partial hepatic ischemia, the clip was removed to start the process of reperfusion. The mice in the sham-operated group underwent a surgical technique that was identical to that of the experimental group, with the only difference being that deliberate obstruction of blood arteries was not performed. The mice were subjected to euthanasia at predetermined time intervals subsequent to reperfusion to obtain blood and tissue samples for subsequent analysis.

### Cell culture

AML12 cells (cat. no. SCSP-502) were obtained from the Institute of Biochemistry and Cell Biology, Chinese Academy of Sciences. The cells were cultured in a mixture of Dulbecco’s modified Eagle medium and F-12 nutrient mixture (1:1) supplemented with 10% fetal bovine serum, 1% insulin-transferrin-selenium liquid media supplement (Sigma), and 40 ng/mL dexamethasone (Sigma).

To extract primary liver cells, the mice were initially subjected to 5 min of perfusion with a calcium-free HEPES solution, after which an in situ collagenase procedure using type IV collagenase from Sigma was employed. The primary hepatocytes were placed in a centrifuge, where they were separated and isolated at a force of 50 × *g*, the acceleration due to gravity, and this process lasted for 5 min. The resulting supernatants were subsequently subjected to centrifugation for a second time at a force of 500 times the acceleration due to gravity for 10 min. The primary hepatocytes were resuspended in a 10 mL mixture of Dulbecco’s modified Eagle medium (DMEM) and 90% Percoll. The cells were seeded into 6-well culture dishes with DMEM/F12 supplemented with 10% fetal bovine serum (FBS). The samples were subsequently incubated in a humidified incubator containing 5% carbon dioxide (CO_2_), and the temperature was set at 37 °C.

To establish an IRI model in an artificial environment, the cellular samples were exposed to hypoxia/reoxygenation (H/R) conditions. The primary hepatocytes and AML12 cells were exposed to a low-oxygen environment with only a 1% oxygen concentration in glucose-free DMEM for a period of 6 h. The cells were subsequently grown under normal atmospheric pressure for various durations as needed.

### In vivo adenovirus injection and treatment

The mice were injected with 100 µL of MAFF-specific recombinant adenovirus via the tail vein using either rAd-OEMAFF (1.0 × 10 [11] viral genomes (vg)/ml) or rAd-shMAFF (1.0 × 10 [11] viral genomes (vg)/ml), along with the corresponding empty vector recombinant adenovirus (NC). Subsequently, 3 days later, a liver IRI model was established in these animals.

### In vitro transfection and treatment

The plasmid was incubated for 15 min with transfection reagents (Invitrogen, Lipofectamine^TM3000^), followed by a subsequent experiment in which the cells were incubated at 37 °C for 48 h with the indicated transfection mixture. The relevant sequences are outlined in Supplementary Table 1.

### Liver function evaluation

Serum aspartate aminotransferase (AST) and alanine aminotransferase (ALT) levels were determined using commercially available kits obtained from Nanjing Jiancheng Bioengineering Institute, China.

### Histology staining

Histopathological analysis was conducted using hematoxylin and eosin (H&E) staining (Solarbio). In addition, a commercially available in situ apoptosis detection kit (Beyotime Biotechnology C1090) was used to conduct terminal deoxynucleotidyl transferase dUTP nick end labeling (TUNEL) on paraffin-embedded liver tissue slices following the instructions provided by the manufacturer.

### Real-time quantitative polymerase chain reaction (RT-qPCR)

The RNA extraction from liver tissue or cells was performed using TRIzol reagent (Takara) following the guidelines provided by the manufacturer. The mRNA was reverse transcribed using PrimeScript^™^ RT Master Mix (Takara). Quantitative real-time PCR was performed on an ABI 7500 (Thermo) with SYBR® PremixExTaq™ II (Takara). β-Actin served as the internal control to normalize mRNA expression, and the fold change values were determined using the 2^−ΔΔCt^ method. The primer sequences are presented in Supplementary Table 2.

### Luciferase reporter assay

A total of ten [4] cells were evenly distributed in a 96-well plate. The cells were subsequently transfected with X-tremeGENE9TM DNA transfection reagent containing either the HRE or the mutated pGL4 firefly luciferase vector, as well as the IL11 promoter with MARE. In addition, 5 ng of Renilla luciferase vector was cotransfected into the cells (Millipore Sigma, no. 6,365,787,001). The cells were subjected to H/R for 48 h after transfection.

### Immunohistochemistry (IHC)

After boiling in citrate buffer at a temperature above 95 ℃ for 20 min, the liver sections were measured at 5 μm, dewaxed, hydrated, and subjected to antigen retrieval, after which they were subsequently stained with an immunohistochemical detection kit (PV-9000, ZSGB-BIO, Beijing, China) in accordance with the instructions provided by the manufacturer. The sections were treated with MAFF (12,771–1-AP, Proteintech, 1:100), Ly6g (ab303467, Abcam, 1:100), and P-STAT3 (ab76315, Abcam, 1:100) at 4 °C overnight. The sections were subsequently stained with a diaminobenzidine (DAB) solution before being captured using light microscopy (Olympus, Japan).

### Immunofluorescence (IF)

The cells were treated with a 4% paraformaldehyde solution for 20 min. For the tissue samples, liver sections that were covered with paraffin (with a thickness of 5 μm) underwent a process of dewaxing, hydration, and heat retrieval using ethylene diamine tetra-acetic acid (EDTA) antigen repair solution. The samples were subsequently exposed to a Triton X-100 solution (Beyotime, China) for 15 min at room temperature to facilitate permeabilization. The samples were subsequently blocked with a 3% BSA solution for 1 h. The samples were treated overnight at 4 °C with primary antibodies against MAFF (12,771–1-AP, Proteintech, 1:100), F4/80 (14–4801-85, Thermo Fisher Scientific, USA, 1:50), Bach1 (66,762–1-Ig, Proteintech, 1:100), and CD11b (ab52478, Abcam, 1:100). The secondary antibodies used in this study included Cy3-conjugated goat anti-rabbit (SA00009-2, Proteintech, 1:100), FITC-conjugated goat anti-rabbit (SA00003-2, Proteintech, 1:100), Cy3-conjugated goat anti-rat (A10522, Thermo Fisher Scientific, USA, 1:500), and FITC-conjugated goat anti-mouse (SA00003-1, Proteintech, 1:100) antibodies. Diamidine phenylindole (DAPI) was used to stain the cell nucleus. Images were obtained using fluorescence microscopy equipment manufactured by Olympus in Japan.

### Co-immunoprecipitation (Co-IP)

Protein interactions were confirmed using coimmunoprecipitation (Co-IP). Initially, the cellular lysates were placed on a rotator, where they were incubated with anti-MAFF, anti-Bach1, or rabbit immunoglobulin G (IgG) (4.0 µg) at 4 °C overnight. The suspension mentioned earlier was subjected to incubation at 4 °C with continuous rotation for 2 h in the presence of protein A/G magnetic beads. Following the washing step, the antigen was effectively removed by boiling in SDS‒PAGE protein loading buffer (2X) for 10 min. The mixture of bantigen‒antibody‒magnetic beads was further subjected to western blotting (WB) analysis.

### RNA sequencing and CUT&Tag

The primary hepatocytes were oxygenated for 6 h and reoxygenated for 6 h. The cells were subsequently collected for relevant detection. Supplementary Data 1 shows the specific steps.

### Chromatin immunoprecipitation (ChIP)-PCR

We extracted primary liver cells and established an H/R model. Chromatin immunoprecipitation was performed using a chromatin immunoprecipitation kit (P2078, Beyotime). In brief, protein‒DNA complexes from treated cells were crosslinked with formaldehyde at room temperature for 10 min. The DNA extracted from disrupted cellular material was sonicated to generate approximately 500 base pair (bp) fragments. The resulting lysate was incubated with a Maff-specific antibody and precipitated using protein G magnetic beads. After three washes, the DNA‒protein complex was reverse crosslinked, and genomic DNA was extracted using a DNA purification kit (D0033, Beyotime) and eluted in buffer solution. PCR amplification was performed using 0.5 μg of DNA as a template with the primers listed in Supplementary Table 3. The separation of the PCR products was performed on a 2.0% agarose gel, and visualization was performed via ethidium bromide staining and ultraviolet (UV) light illumination.

### Western blotting

RIPA lysis buffer supplemented with 1% phenylmethylsulfonyl fluoride (PMSF) and a protein phosphatase inhibitor was used to extract proteins. Subsequently, the lysates were supplemented with 5 × SDS loading buffer at a volume equivalent to one fourth of the lysate volume and boiled for 10 min. The experiment involved the use of equal protein samples that were loaded into SDS–polyacrylamide gels with varying concentrations (8%, 10%, or 12%). These gel samples were subsequently subjected to electrophoresis, and the proteins obtained were then transferred onto polyvinylidene fluoride (PVDF) membranes. Next, the membranes were subjected to a 2-h blocking process with 5% skim milk. After the blocking process, the membranes were incubated with primary antibodies at 4 °C overnight. The primary antibodies used in this study included anti-MAFF (12,771–1-AP, Proteintech, 1:100), anti-Bcl2 (3498 T, Cell Signaling Technology, 1:1000), anti-Bax (2772 T, Cell Signaling Technology, 1:1000), anti-cleaved-caspase-3 (9664 T, Cell Signaling Technology, 1:1000), anti-STAT3 (ab68153, Abcam, 1:2000), anti-P-STAT3 (ab76315, Abcam, 1:2000), anti-Bach1 (14,018–1-AP, Proteintech, 1:1000), and anti-β-actin (AB0035, Abways, 1:5000) antibodies. Following a systematic washing process using Tris-buffered saline with Tween (TBST) on the following day, the membranes were incubated with a secondary antibody linked to horseradish peroxidase (HRP) (1:5000) for 1 h at 37 °C. Enhanced electrochemiluminescence (ECL) reagent was used to detect the immunoreactive bands, whereas β-actin served as a reference for standardizing the relative protein expression.

### Flow cytometry

Flow cytometry was used to assess the extent of cellular apoptosis. The cells were enzymatically dissociated using trypsin, followed by rinsing with phosphate-buffered saline (PBS). The cells were subsequently resuspended in PBS. To evaluate apoptosis, Annexin V-fluorescein isothiocyanate (FITC) and propidium iodide (PI) were administered to the cells. Ultimately, flow cytometry was utilized to examine the cells.

### Statistical analysis

The statistical analyses were conducted using SPSS 22.0 software. Student’s *t*-test, one-way analysis of variance, the Mann–Whitney *U* test, and Dunn’s multiple comparison test were used for statistical analysis, where *P < 0.05, **P < 0.01, and ***P < 0.001.

## Results

### MAFF may be involved in hepatic IRI

**Fig. 1:**
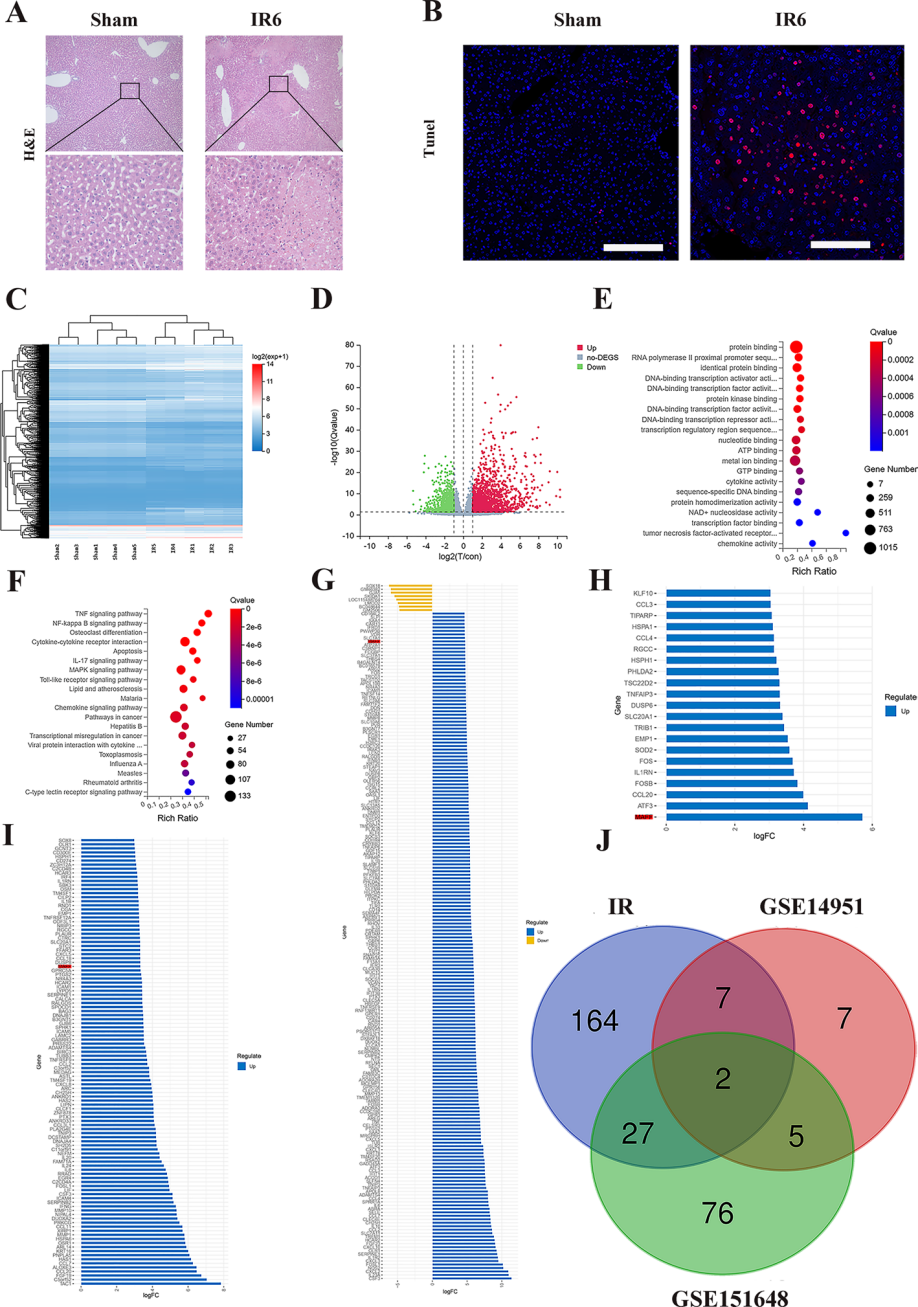
IRI-related genes were identified by sequencing and database analysis after modeling. **A** H&E staining was used to evaluate areas of necrosis; scale bar = 20 and 100 μm. **B** TUNEL staining was performed to detect cell death; scale bar = 50 μm. **C** and **D** Heatmap and volcano map showing the differentially expressed genes (DEGs) identified after IRI (│logFC│ > 1, *p* < 0.05). **E** and **F** GO and KEGG analyses of the differentially expressed genes. **G** Abundance map of the top 200 DEGs according to the sequencing results. **H** Abundance map of the DEGs in the GSE14951 dataset (|logFC│ > 2, *p* < 0.05). **I** Abundance map of the DEGs in the GSE151648 dataset (|logFC│ > 2, *p* < 0.05). **J** The Venn diagram illustrates the overlap between the sequencing screening and analysis of liver transplant databases

Initially, we developed a liver IRI model. As depicted in Supplementary Fig. 1, ALT and AST levels increased early within 1 h after reperfusion and peaked at 6 h. Therefore, we selected 6 h of reperfusion for subsequent high-throughput sequencing studies. H&E and TUNEL staining of the tissue sections revealed significant pathological damage following liver IRI (Fig. 1A, B). Subsequent high-throughput sequencing analysis revealed the downregulation of 223 genes and the upregulation of 801 genes following IRI (Fig. 1C, D, Supplementary Table 4). Gene ontology (GO) analysis revealed the involvement of numerous biological processes in IRI, including protein binding, adenosine triphosphate (ATP) binding, guanosine triphosphate (GTP) binding, nucleotide nicotinamide adenine dinucleotide (NAD^+^) nucleosidase activity, and transcription factor binding (Fig. 1E). Kyoto Encyclopedia of Genes and Genomes (KEGG) analysis revealed that liver IRI involves complex signaling networks, including the tumor necrosis factor (TNF) signaling pathway, the nuclear factor-κB (NF-κB) signaling pathway, and apoptosis (Fig. 1F). We selected the top 200 genes with the most significant expression differences for further analysis (Fig. 1G).

For further analysis, five pairs of tissue samples were randomly selected from the GSE14951 dataset before and after liver transplantation and reperfusion. In addition, 23 pairs of tissue samples exhibiting notable pathological injuries before and after liver transplantation and reperfusion were randomly selected from the GSE151648 dataset. We identified 21 and 110 DEGs in the GSE14951 and GSE151648 datasets, respectively (Fig. 1H–I). A comprehensive review of these datasets revealed that only two genes, MAFF and IL1RN, exhibited significant differences across all three datasets (Fig. 1J). The role of IL1RN in hepatic IRI has been previously elucidated [16], so we chose MAFF for follow-up studies.

### MAFF expression is increased in hepatic IRI

**Fig. 2:**
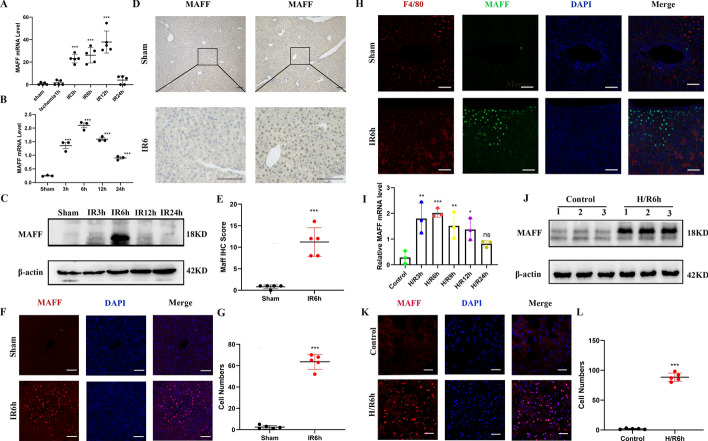
MAFF expression is upregulated during hepatic IRI. **A** MAFF mRNA levels increased, as indicated by the database (PRJNA407106). **B** RT-qPCR revealed a steady increase in MAFF mRNA levels. **C** WB analysis was used to evaluate and quantify MAFF protein levels in the mice. **D**–**G** MAFF expression was observed using IHC and IF (*n* = 5); scale bars = 50 μm and 20 μm. Immunofluorescence indicated that MAFF was expressed mainly in hepatocytes; scale bar = 50 μm. **I** and **J** MAFF mRNA and protein levels in hepatocytes were examined using RT-qPCR at different time points (*n* = 3) and WB analysis after 6 h of H/R, respectively. **K** and **L** Immunofluorescence was used to observe the expression of MAFF in hepatocytes following H/R treatment (*n* = 5); scale bar = 50 μm.

To investigate whether hepatic IRI is associated with MAFF expression, we initially analyzed MAFF expression using data from a publicly available database (PRJNA407106). The findings revealed a gradual increase in the expression of the MAFF gene at 3, 6, 12, and 24 h after I/R injury (Fig. 2A). We discovered that MAFF expression increased steadily at 3, 6, 12, and 24 h during the reperfusion period in a hepatic IRI model in C57BL/6 mice, with the most significant increase found at 6 h (Fig. 2B, C). IHC and IF analyses further confirmed a noticeable increase in MAFF expression in liver tissues (Fig. 2D–G), particularly in severely damaged areas after hepatic IRI (Supplementary Fig. 2). In addition, our immunofluorescence experiments revealed that MAFF does not colocalize with F4/80, a marker of hepatic parenchymal cells (macrophages) (Fig. 2H). Hence, we propose that MAFF is expressed predominantly in hepatic parenchymal cells and is localized within the cell nucleus of hepatocytes (Fig. 2K).

To conduct a more comprehensive investigation of the alterations in MAFF expression within hepatocytes, primary hepatocytes were subjected to H/R stimulation. Consistent with the in vivo findings, MAFF mRNA (Fig. 2I) and protein (Fig. 2J) levels significantly increased after H/R stimulation of cultured hepatocytes. This phenomenon was further elucidated using immunofluorescence of primary hepatocytes following H/R stimulation (Fig. 2K–L). These findings indicate a prospective relationship between MAFF and hepatic IRI, suggesting that MAFF may play a significant role in this process.

### MAFF protects the liver during hepatic IRI

**Fig. 3:**
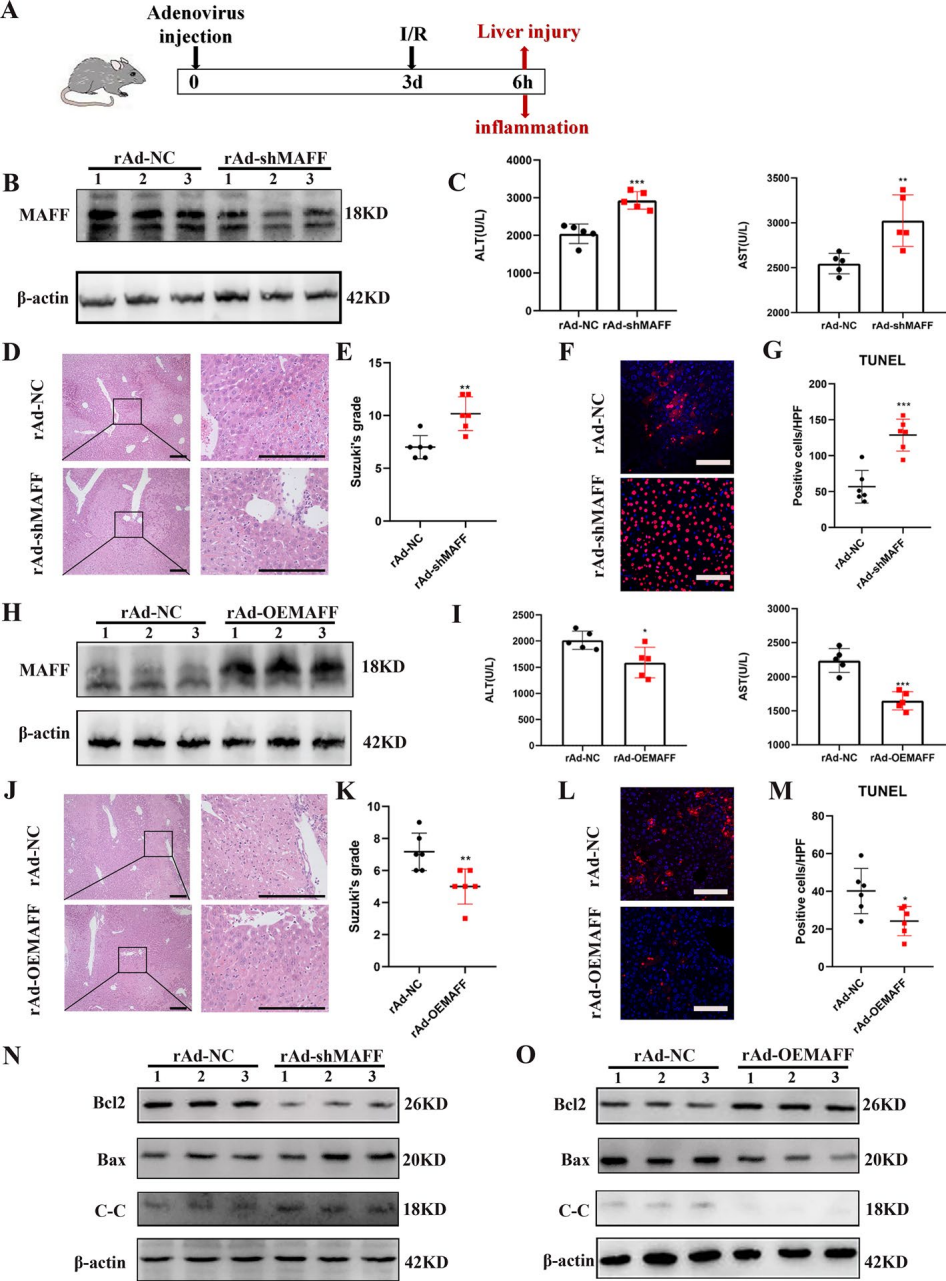
MAFF protects against liver damage. **A** Hepatic IRI model. **B** Expression of MAFF protein in the livers of rAd-NC group and rAd-shMAFF group. **C** The serum levels of ALT and AST were identified in both rAd-NC and rAd-shMAFF mice at 6 h after hepatic IRI (*n* = 5). **D** and **E** At 6 h post hepatic IRI, liver tissue samples from mice treated with rAd-NC and rAd-shMAFF were analyzed using H&E staining (*n* = 5); scale bar = 100 μm. **F** and **G **TUNEL staining was used to evaluated cell death of ischemic liver sections (*n* = 5); scale bar = 50 μm. **N** The expression of cleaved-caspase-3, Bax, and Bcl2 in the livers of both rAd-NC and rAd-shMAFF mice was analyzed using WB. **H** Expression of MAFF protein in the hepatic tissues of mice administered with rAd-NC and rAd-OEMAFF. **I** ALT and AST were identified in both rAd-NC and rAd-OEMAFF mice after hepatic IRI (*n* = 5). **J** and **K** At 6 h after hepatic IR, H&E staining demonstrated necrotic regions of liver tissue from rAd-NC and rAd-OEMAFF mice (*n* = 5); scale bar = 100 μm. **L** and **M** TUNEL staining was performed on ischemic liver slices obtained from mice belonging to the specified groups (*n* = 5); scale bar = 50 μm. The expression of cleaved-caspase-3, Bax, and Bcl2 in the livers of both rAd-NC and rAd-OEMAFF mice was analyzed using WB

To investigate the impact of MAFF on hepatic IRI, we generated specific rAd-MAFF mice and subjected them to hepatic IRI (Fig. 3A). WB analysis revealed a significant reduction in MAFF expression in the liver tissues of the mice treated with rAd-shMAFF (Fig. 3B). We evaluated hepatic damage by quantifying the serum AST and ALT concentrations and assessing the degree of necrotic tissue. Compared with those in the rAd-NC group, serum AST and ALT levels were notably elevated when MAFF expression was reduced (Fig. 3C). In addition, the extent of the necrotic region in the rAd-shMAFF group was significantly greater than that in the rAd-NC group, as shown by H&E staining (Fig. 3D and E). Apoptosis plays a vital role in the progression of hepatic ischemia‒reperfusion injury [17]. TUNEL staining revealed an increased number of apoptotic cells in rAd-shMAFF mice (Fig. 3F and G). In addition, the rAd-shMAFF group presented increased levels of proapoptotic proteins, including Bax and cleaved-caspase-3 (C–C), whereas a significant decrease in the expression of the antiapoptotic protein Bcl2 was observed compared with the control group (Fig. 3N). In contrast, MAFF overexpression alleviated liver damage, tissue necrosis, and cellular apoptosis (Fig. 3H–M, O). Therefore, we suggest that MAFF could protect the liver during hepatic IRI.

### MAFF reduces the inflammatory response in hepatic IRI

**Fig. 4:**
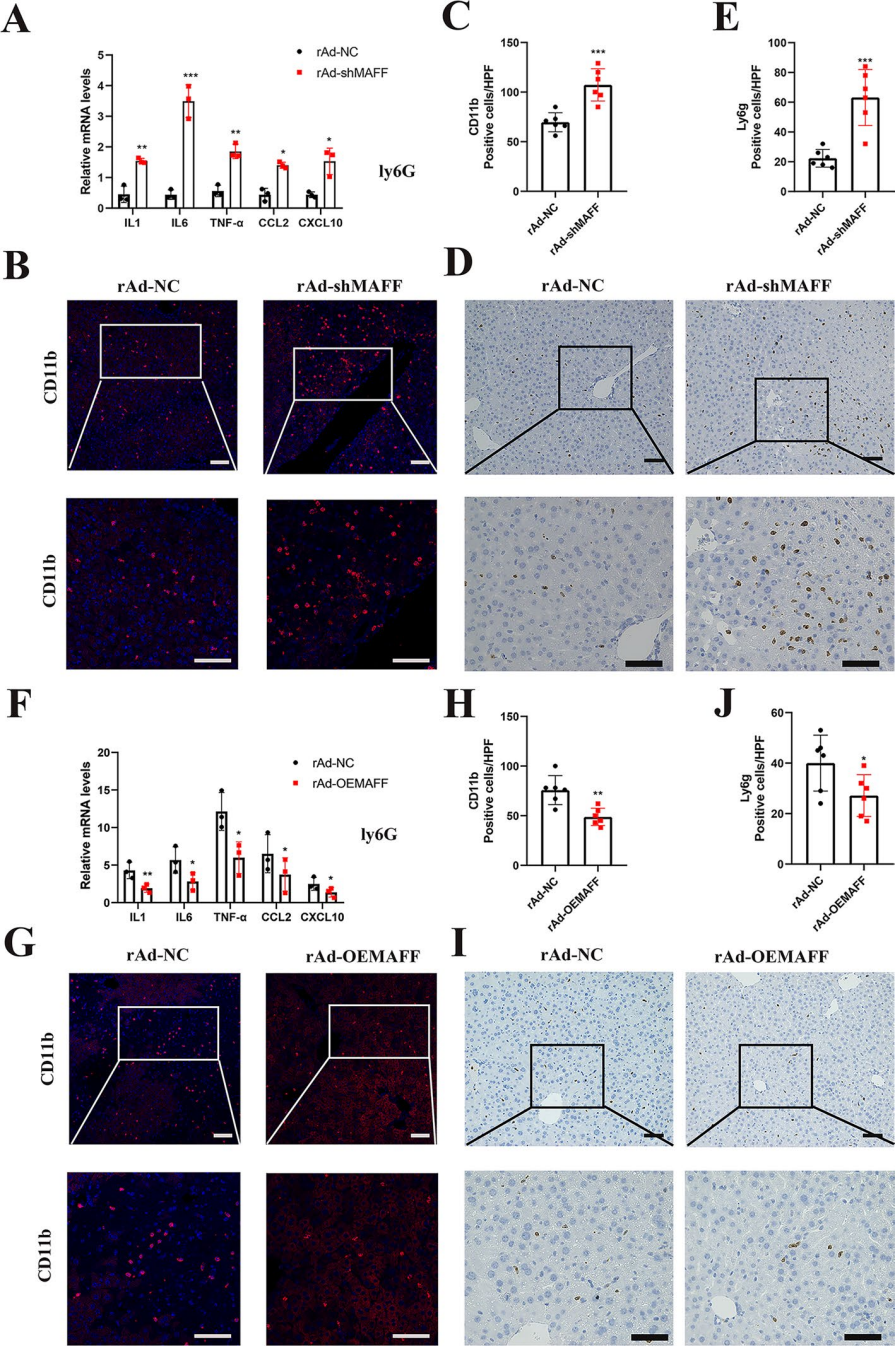
MAFF relieves inflammation in hepatic IRI. **A** At 6 h after hepatic IRI, the liver tissues of both rAd-NC and rAd-shMAFF mice were analyzed to assess mRNA levels of inflammatory cytokines and chemokines (*n* = 3). **B** and **C** Immunofluorescence was performed to detect CD11b-positive inflammatory cells (red) in ischemic liver sections obtained from mice in the specified experimental groups (*n* = 6); scale bar = 50 and 100 μm. **D** and **E** IHC was performed to assess Ly6G expression in ischemic liver sections obtained from mice in the specified experimental groups (*n* = 6); scale bar = 100 μm. **F** At 6 h after hepatic IRI, the liver tissues of both the rAd-NC and the rAd-OEMAFF mice were analyzed to assess mRNA levels of inflammatory cytokines and chemokines (*n* = 3). **G** and **H** Immunofluorescence staining was used to identify inflammatory cells expressing CD11b (red) in liver sections from the mice assigned to the designated experimental groups (*n* = 6); scale bars = 50 and 100 μm. **I** and **J** IHC was conducted to evaluate the presence of Ly6G in liver sections affected by ischemia, using samples obtained from mice assigned to specific experimental groups (*n* = 6); scale bar = 100 μm

Hepatic IRI is significantly influenced by activated inflammation [18], and an effective treatment for IRI involves reducing the inflammatory response. Thus, we also investigated whether MAFF affects the inflammatory reactions caused by hepatic IRI. First, at 6 h after IRI, the livers of the rAd-shMAFF mice presented substantially higher mRNA levels of C-X-C motif chemokine 10 (Cxcl10), chemokine ligand 2 (Ccl2), IL-6, IL-1, and TNF-α compared with rAd-NC mice (Fig. 4A). The immunofluorescence results indicated that infiltration of liver tissues by cells expressing CD11b was significantly greater in the rAd-shMAFF group (Fig. 4B and C). Furthermore, IHC revealed a noteworthy increase in Ly6G expression in liver tissues from the rAd-shMAFF group compared with those from the control group (Fig. 4D and E). In contrast, inflammatory cell infiltration and the levels of inflammatory cytokines and chemokines were lower in the rAd‐OEMAFF mice compared with the rAd-NC mice (Fig. 4F–J). These results suggest that MAFF reduces inflammatory reactions during hepatic IRI.

### RNA sequencing and CUT&Tag analysis of MAFF-mediated gene regulation at the genome level

**Fig. 5:**
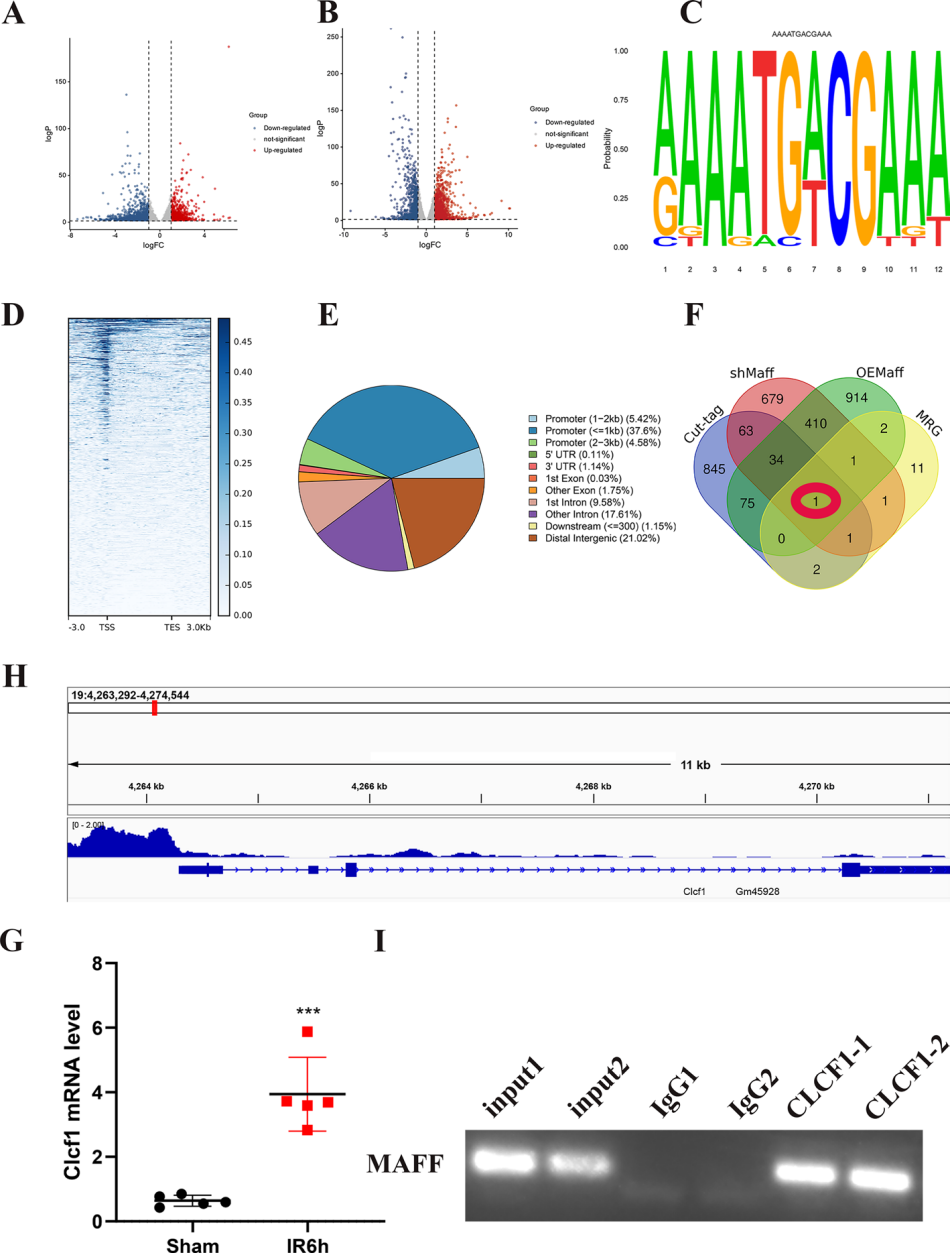
Identification of MAFF target genes using genome-wide analysis. **A** Volcano plot showing gene expression after interference with MAFF (│logFC│ > 1, *p* < 0.05). **B** Volcano plot showing gene expression after MAFF overexpression (│logFC│ > 1, *p* < 0.05). **C** The motif algorithm was used to identify the MAFF-binding motif on the basis of MARE. **D** Heatmap showing that MAFF-binding sites are located mainly in the promoter region. **E** Chromosomal coordinates of MAFF-binding sites. **F** Integration of RNA sequencing, CUT&Tag methods and MRG to identify genes under the control of MAFF genes during IRI. **G** RT-qPCR was used to assess CLCF1 mRNA expression levels following IRI. **H** The CUT&Tag sequencing results revealed an abundance of DNA sequence tags. **I** ChIP-PCR showed that MAFF binds to CLCF1

To investigate the underlying mechanism of IRI induced by MAFF, we conducted RNA sequencing analysis on liver tissues collected from mice treated with rAd-shMAFF, rAd-NC (shMAFF), rAd-OEMAFF, or rAd-NC (OEMAFF) after undergoing liver IRI. The volcano plot revealed that a majority of the genes were differentially expressed (Supplementary Tables 5 and 6, Fig. 5A and B). MEME analysis revealed a motif enriched in MAFF that was similar to the consensus MARE sequence (Fig. 5C). The heatmap shown in Fig. 5D indicates that most MAFF-binding sites were located within the promoter region. Global analysis of CUT&Tag data revealed that 46.7% of MAFF binding occurred within the promoter region, whereas 28.89% of MAFF binding was observed within intron regions (Fig. 5E). Hence, in the following investigation, we opted for genes exhibiting reduced expression in the knockdown group and genes displaying enhanced expression in the overexpression group. According to Moritz’s findings, MAFF potentially exerts control over a set of 19 genes located downstream in mice [19] (Supplementary Table 7). Using a combination of RNA sequencing, CUT&Tag profiles, and MAFF-related genes (MRGs), our study identified CLCF1 as a potential gene that is directly bound and controlled by MAFF (Fig. 5F). RT-qPCR analysis results revealed significant upregulation of CLCF1 following hepatic ischemia‒reperfusion injury (Fig. 5G). Further examination of MAFF-binding peaks in the target gene promoters using the UCSC Genome Browser revealed a conspicuous peak located within the promoter region of CLCF1, which encompasses a MARE/ARE sequence. These findings indicate that CLCF1 transcription is directly controlled by MAFF (Fig. 5H). Finally, repeated use of ChIP-PCR further confirmed that the MAFF protein binds to the CLCF1 promoter region (Fig. 5I).

### CLCF1 expression is regulated by the MAFF protein

**Fig. 6:**
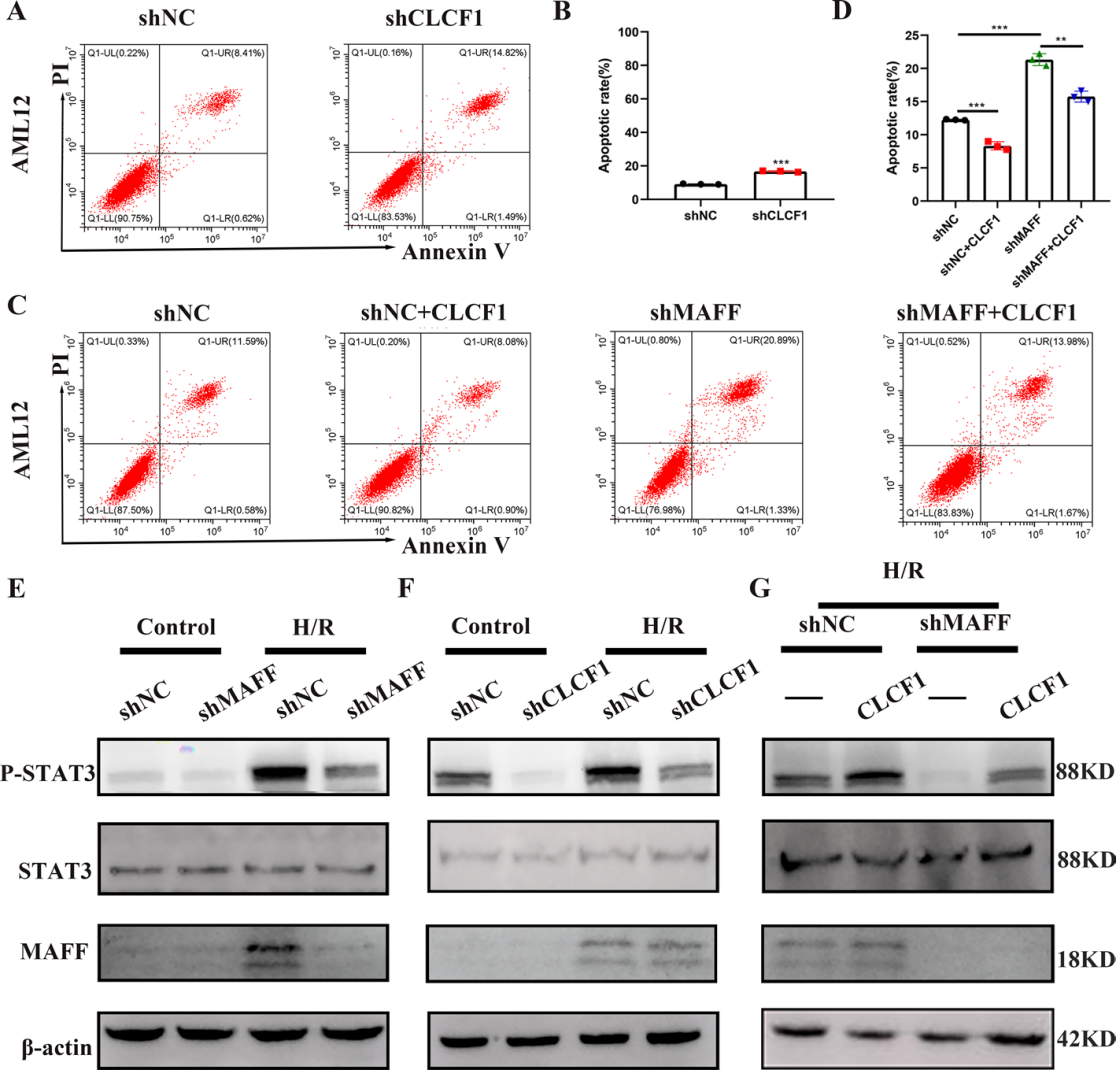
Hepatocyte apoptosis is regulated by the MAFF-mediated CLCF1 pathway. **A**–**B** Flow cytometry was used to evaluate cell apoptosis in hepatocytes exposed to shCLCF1 (*n* = 3). **C**–**D** The impact of CLCF1 on AML12 cell apoptosis both with and without MAFF knockdown, as determined using flow cytometry (*n* = 3). **E** WB was used to assess STAT3 activation in AML12 cells subjected to either normoxia or H/R in the shNC and shMAFF groups. **F** WB was used to assess STAT3 activation in AML12 cells subjected to either normoxia or H/R in the shNC group and shCLCF1 group. **G** WB was used to assess STAT3 activation in AML12 cells under H/R stimulation following the addition of exogenous CLCF1 in the shNC and shMAFF groups

CLCF1, a member of the IL-6 family, is a cytokine that plays a role in the processes of inflammation [20]. To determine the importance of CLCF1 in MAFF-mediated cell death, shRNA was used to silence CLCF1 in AML12 cells. A decrease in CLCF1 expression resulted in a considerable increase in apoptosis (Fig. 6A and B). In contrast, the introduction of exogenously produced CLCF1 effectively mitigated the increased apoptosis observed in MAFF-knockdown cells, indicating that MAFF plays a regulatory role in cell apoptosis through the CLCF1 pathway (Fig. 6C and D). The cellular response to CLCF1 is mediated by STAT3 activation, which is thought to be involved in apoptosis [21, 22]. We found that the induction of phospho-STAT3 was suppressed upon the inhibition of MAFF or CLCF1 in AML12 cells (Fig. 6E and F). In contrast, the addition of exogenous CLCF1 protein to MAFF-knockdown cells restored STAT3 activation (Fig. 6G). These results indicate that STAT3 pathway activation by MAFF is mediated through the direct regulation of CLCF1.

### MAFF regulates cell apoptosis by a BACH1-dependent mechanism

**Fig. 7:**
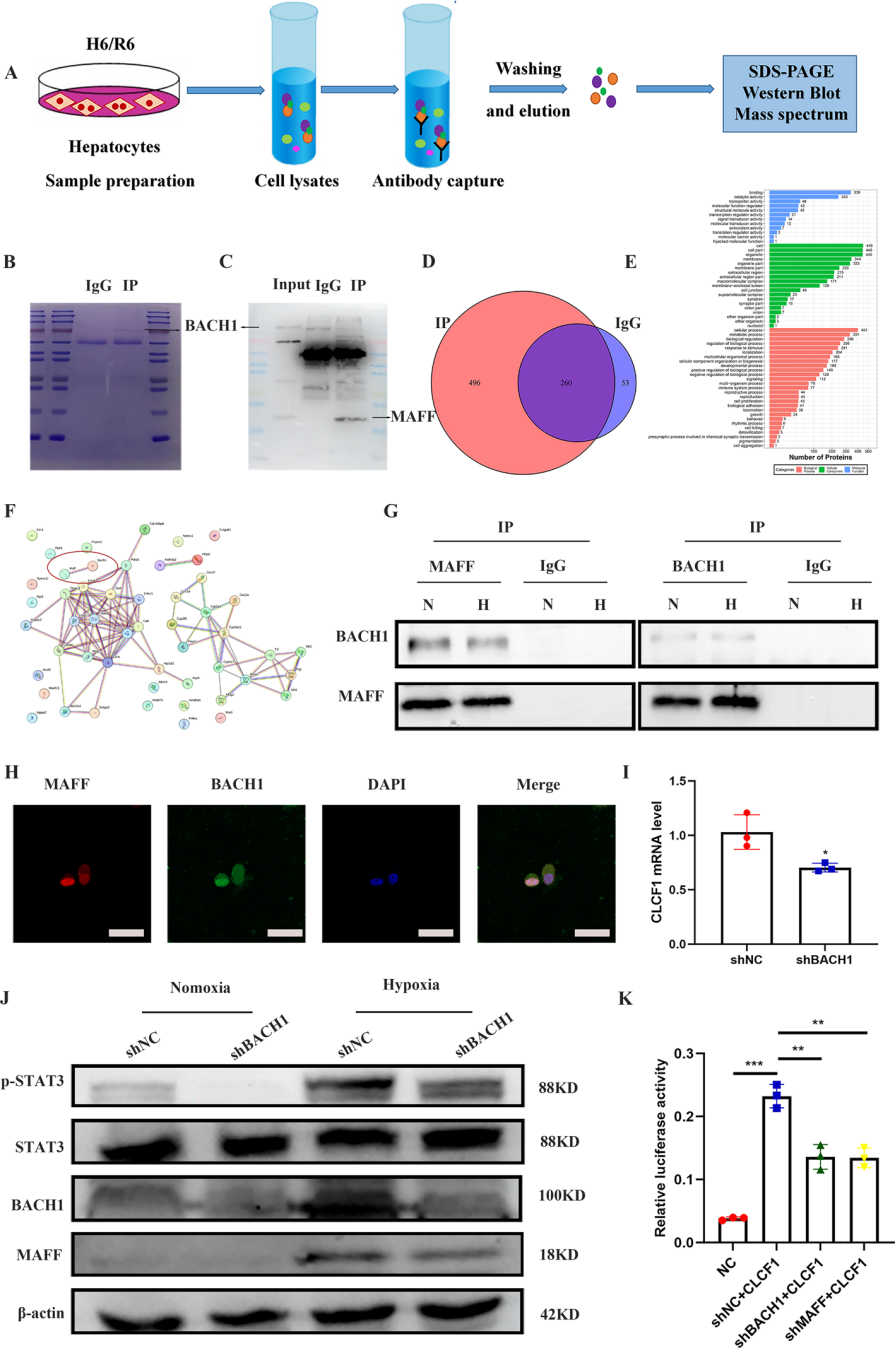
MAFF binding to BACH1 facilitates the activation of the CLCF1/STAT3 signaling pathway. **A–E** Following immunoprecipitation (IP) with MAFF antibody or IgG (negative control antibody), samples collected from hepatocytes were subjected to electrophoresis. Subsequently, Coomassie brilliant blue staining was performed. This staining technique was employed after the samples were subjected to H/R. The present study employed mass spectrum analysis to examine IP-MAFF and IP-IgG lysates. A Venn diagram was generated, and GO analysis was performed. **F** Interactions among 50 proteins. **G** Co-IP of MAFF and BACH1 in hepatocytes after H/R. **H** Immunofluorescence staining of MAFF, BACH1, and DAPI in hepatocytes after H/R; scale bar = 20 μm. **I** RT-qPCR was used to assess CLCF1 levels (*n* = 3). **J** The effect of BACH1 on the STAT3 pathway in liver cells was evaluated using WB analysis after suppressing BACH1 expression. **K** A luciferase test was conducted utilizing the pGL4-luciferase vector. The transcriptional activity of the discovered MARE was reduced upon BACH1 or MAFF knockdown (*n* = 3)

We propose that the transactivation or repression mediated by MAFF is contingent upon its specific binding partner. To identify the proteins that interact with MAFF and play a role in cell death, we utilized techniques involving affinity purification and mass spectrometry (AP-MS) (Fig. 7A–C). Supplementary Table 8 shows that 313 proteins were identified in the IP-IgG group, whereas 756 proteins were identified in the IP-MAFF group. There were 496 IP-MAFF group-specific proteins, as shown in the Venn diagram (Fig. 7D). According to the GO analysis, MAFF functions primarily in cellular processes, with its associated molecules representing its most significant molecular functions (Fig. 7E). The mass spectrometry results were subsequently examined to identify the primary MAFF interactions in AML12 cells, which revealed BACH1 as its exclusive binding partner among the top 50 proteins (Fig. 7F). BACH1, a genetically distant CNC protein, acts as a repressor by regulating antioxidant genes through competitive interactions with CNC proteins for binding to MARE or ARE sites [8]. Co-IP analysis results supported the association between BACH1 and MAFF (Fig. 7G). Immunofluorescence revealed significant colocalization of MAFF and BACH1 within the nucleus (Fig. 7H). Downregulation of CLCF1 levels and phospho-STAT3 expression under hypoxia was observed in AML12 cells when BACH1 was suppressed using shRNA (Fig. 7I–J). To determine how MAFF and BACH1 interact with the CLCF1 promoter, we conducted luciferase reporter assays. MARE transcriptional activity decreased when BACH1 or MAFF was knocked down using the same region that was cloned and inserted into the pGL4 luciferase vector (Fig. 7K). Therefore, these findings indicate that CLCF1 is directly regulated by MAFF and BACH1 and that it plays an active role in regulating cell death.

### MAFF regulates hepatocyte apoptosis by the CLCF1/STAT3 signaling pathway

**Fig. 8:**
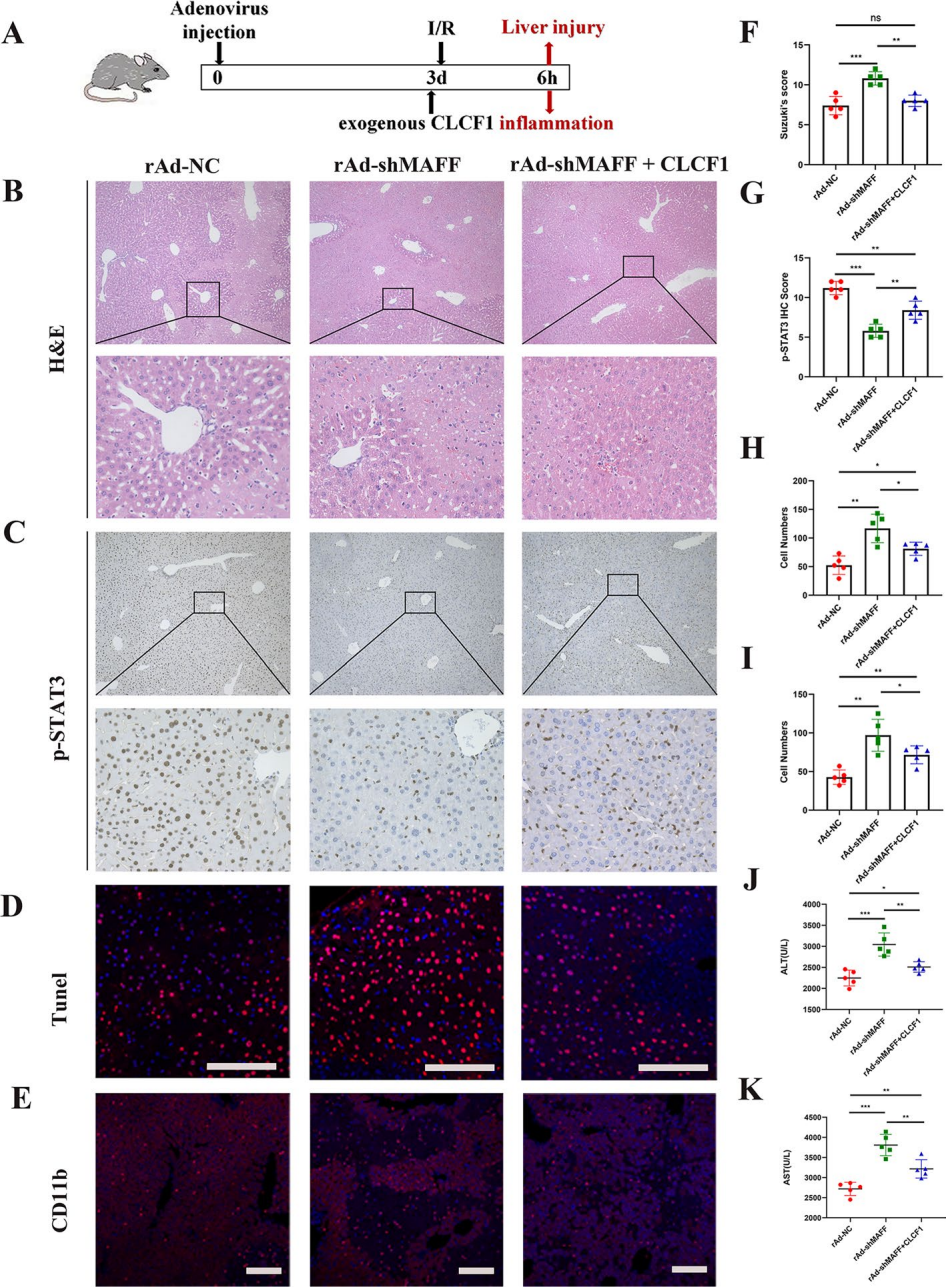
Liver IRI is protected in vivo by MAFF via the CLCF1/STAT3 pathway. **A** Hepatic IRI model. **B** and **F** Histological analysis using H&E staining revealed the presence of necrotic areas in the liver tissue (*n* = 5); scale bars = 100 μm and 20 μm. **C** and **G** IHC was conducted to evaluate P-STAT3 expression in liver sections (*n* = 5); scale bars = 100 μm and 20 μm. **D** and **H** TUNEL staining and statistical analysis were performed on ischemic liver sections obtained from the mice; scale bar = 50 μm. **E** and **I** Immunofluorescence staining was used to identify inflammatory cells expressing CD11b (red) in liver slices affected by ischemia (*n* = 5); scale bar = 100 μm. **J** and **K** ALT and AST levels in the blood were assessed in the rAd-NC, rAd-shMAFF, and rAd-shMAFF + CLCF1 groups (*n* = 5)

To determine how MAFF and its downstream targets, such as CLCF1/STAT3, are potentially important in hepatic IRI, a recombinant adenovirus specific to MAFF (rAd-shMAFF) was used (Fig. 8A). The rAd-shMAFF group was administered recombinant CLCF1 prior to hepatic ischemia‒reperfusion. The present study demonstrated that the reduction in MAFF expression resulted in increased liver damage, whereas exogenous CLCF1 effectively mitigated the observed experimental outcome (Fig. 8B and F). In addition, an investigation of phosphorylated STAT3 expression in liver tissue was conducted to evaluate how MAFF and CLCF1 impact the STAT3 pathway. We discovered that MAFF suppression leads to a decrease in the production of phosphorylated STAT3. However, exogenous CLCF1 counteracts these inhibitory effects in vivo (Fig. 8C and G). TUNEL (Fig. 8D and H) and IF (Fig. 8E and I) analyses further confirmed that exogenous CLCF1 may mitigate the consequences of MAFF knockdown on apoptosis and the infiltration of inflammatory cells following hepatic ischemia‒reperfusion. These findings indicate that following ischemia‒reperfusion caused by MAFF knockdown, CLCF1 may protect the liver from injury, as evidenced by the observed decreases in AST and ALT serum levels (Fig. 8J and K).

## Discussion

Hepatic IRI plays a crucial role in the morbidity and mortality rates associated with liver resection and transplantation procedures [23]. In this study, we identified MAFF as a promising candidate for therapeutic intervention in hepatic IRI. Our findings demonstrated the upregulation of MAFF expression in hepatocytes following hepatic IRI. Suppression of MAFF expression exacerbated hepatic IRI, whereas MAFF overexpression protected against IRI. The interaction between MAFF and BACH1 involves their binding, resulting in the upregulation of the downstream gene CLCF1 and the subsequent activation of the STAT3 signaling pathway. Our findings suggest that MAFF plays a significant role in modulating hepatic IRI regulation.

MAFF, a member of the bZIP family, affects the prognosis of diverse diseases. Deng et al. reported that the suppressed expression of MAFF could enhance fibrosis and the inflammatory response in patients with lupus nephritis [24]. Xie et al. reported that MAFF overexpression inhibited colorectal cancer growth both in vivo and in vitro [25]. Luan et al. reported that MAFF could lead to metabolic reprogramming in pancreatic cancer and increased drug resistance, further resulting in poor patient prognosis [26]. Liang et al. reported that a lower level of MAFF was associated with worse clinical outcomes in patients with lung adenocarcinoma and that MAFF could attenuate tumor cell growth and improve the response to cisplatin or irradiation therapies [27]. In our study, we discovered that MAFF expression is significantly increased in hepatocytes following hepatic IRI. MAFF knockdown increased liver damage, tissue necrosis, cellular apoptosis, and immune cell infiltration after ischemia–reperfusion, whereas MAFF overexpression had the opposite effect.

MAFF has been previously shown to interact with the BACH and CNC protein families to activate or suppress gene transcription [8, 28–31]. In our study, we found that MAFF binds with BACH1 to activate downstream gene transcription in liver IRI. Mechanistically, we identified CLCF1 as a potential gene regulated by MAFF through RNA sequencing and CUT&Tag analysis. We used ChIP-PCR to demonstrate that MAFF protein binds to the CLCF1 promoter region. Therefore, we suggest that MAFF can bind with BACH1 to activate CLCF1 transcription.

CLCF1 is a member of the IL-6 cytokine family, which plays important roles in immune homeostasis, hematopoiesis, inflammation, development, and metabolism [20, 32, 33]. Under certain disease conditions, the production of IL-6 family cytokines increases and is regulated by several signaling pathways, such as the stimulator of interferon genes (STING) [34], phospholipase C (PLC)/protein kinase C (PKC) [35–38], p38/MAPK [37, 39], and p300 [39] signaling pathways. Polonsky et al. reported that CLCF1 was elevated in kidney regeneration and disease [40]. Liu et al. reported that CLCF1 was increased in the liver of diet‐induced nonalcoholic steatohepatitis (NASH) model mice and patients with NASH [41]. Similarly, in our study, we revealed significant upregulation of CLCF1 following hepatic ischemia‒reperfusion injury. Furthermore, we discovered that exogenous CLCF1 effectively alleviated the phenomenon of aggravated apoptosis induced by MAFF knockdown in vitro and mitigated the phenomenon of aggravated liver damage induced by MAFF knockdown in vivo. These findings highlight the important role of CLCF1 in MAFF-mediated liver IRI.

STAT3 is downstream of signaling induced by IL-6 family members [21, 41] and also related to cellular apoptosis processes [22]. Liu et al. reported that CLCF1 promotes tyrosine phosphorylation of STAT3 in primary hepatocytes [41]. Therefore, in our study, we explored the effect of STAT3 downstream of CLCF1. We found that the induction of phospho-STAT3 was suppressed upon the inhibition of MAFF or CLCF1 in AML12 cells. In contrast, the addition of exogenous CLCF1 protein to MAFF-knockdown cells restored STAT3 activation. In vivo, we also discovered that MAFF suppression leads to a decrease in phosphorylated STAT3 and that exogenous CLCF1 counteracts these inhibitory effects. Therefore, we propose that MAFF promotes CLCF1 transcription by interacting with BACH1, activating the downstream STAT3 signaling pathway and ultimately alleviating liver ischemia‒reperfusion injury.

In summary, the significance of MAFF in hepatic IRI has been evaluated. This study demonstrated the hepatoprotective function of MAFF against IRI through the CLCF1/STAT3 pathway. In addition, MAFF serves as a binding partner of CNC and BACH proteins, exerting a significant influence on pathways crucial for hepatocyte apoptosis. Our findings emphasize the pivotal role of MAFF as a transcription factor involved in regulating hepatocyte apoptosis during IRI.

## Conclusions

We suggest that MAFF alleviates hepatic ischemia–reperfusion injury by reducing hepatocyte apoptosis and the inflammatory response through the activation of the CLCF1 and STAT3 signaling pathway, offering valuable insights into the impact of MAFF on liver protection and potential therapeutic targets for liver treatment.

## Supplementary Information

Additional File 1.

Additional File 2.

Additional File 3.

Additional File 4.

Additional File 5.

Additional File 6.

Additional File 7.

Additional File 8.

Additional File 9.

Additional File 10.

Additional File 11.

Additional File 12.

Additional File 13.

## Data Availability

All the data are available upon reasonable request.

## Abbreviations

IRI: Ischemia–reperfusion injury
MAFF: V-maf musculoaponeurotic fibrosarcoma oncogene homolog F
bZIP: Basic leucine zipper
CLCF1: Cardiotrophic factor-like cytokine 1
BACH1: BTB and CNC homology 1
STAT3: Signal transducer and activator of transcription 3
NRL: Neura retinal
ARE: Antioxidant response element
MARE: MAF-recognition element
IL-6: Interleukin-6
PTEN: Phosphatase and tensin homolog
H/R: Hypoxia/reoxygenation
AST: Aspartate aminotransferase
ALT: Alanine aminotransferase
NF-κB: Nuclear factor-κB
TNF: Tumor necrosis factor
ATP: Adenosine triphosphate
GTP: Guanosine triphosphate
NAD: Nucleotide nicotinamide adenine dinucleotide
MRGs: MAFF-related genes
Cxcl10: C-X-C motif chemokine 10
Ccl2: Chemokine ligand 2
