# Supplementary material for: MAFF alleviates hepatic ischemia–reperfusion injury by regulating the CLCF1/STAT3 signaling pathway
Source: Cell Mol Biol Lett. 2025 Apr 1;30:39. doi: 10.1186/s11658-025-00721-x (PMC11963299; doi:10.1186/s11658-025-00721-x)
Supplement: Supplementary file 1 — Additional File 1. [file 11658_2025_721_MOESM1_ESM.pdf]

Full uncut blots

Fig2

C:MAFF

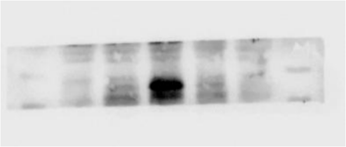

C:actin

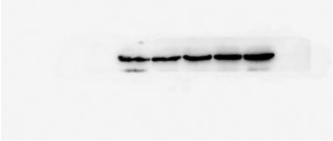

Fig2

J:MAFF

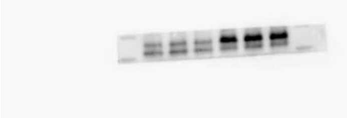

J:actin

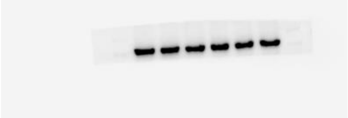

Fig3

B:MAFF

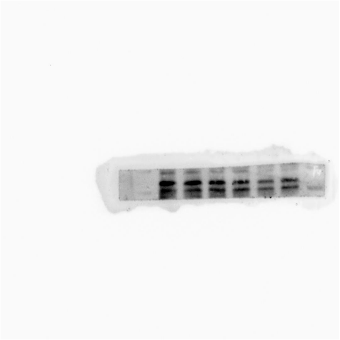

B:actin

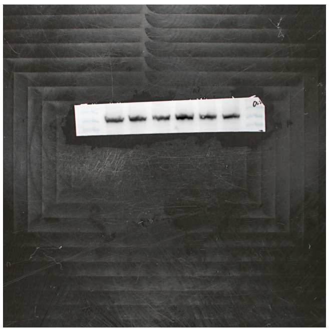

H:MAFF

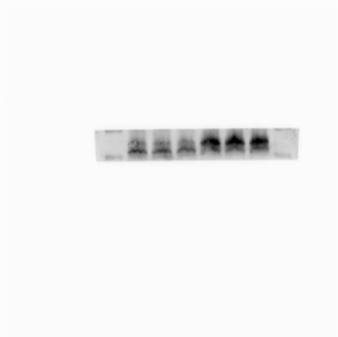

H:β-actin

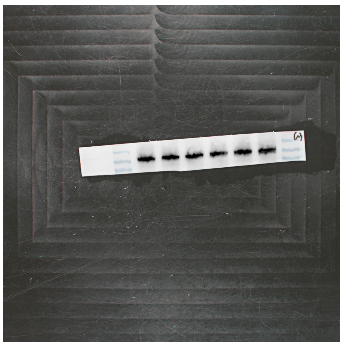

N:β-actin

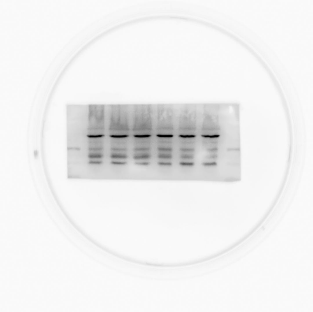

N:C-C

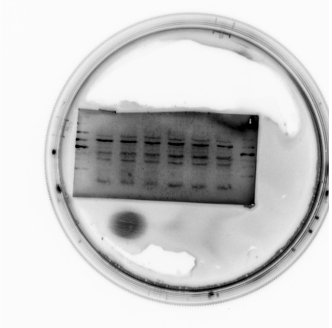

N:Bax

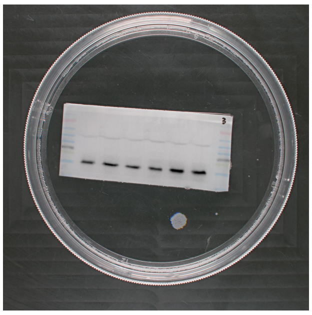

N:Bcl2

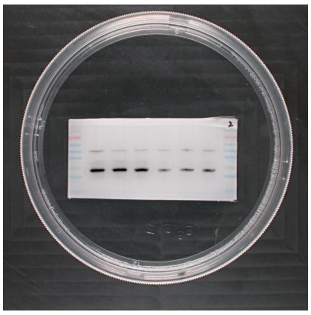

O:C-C

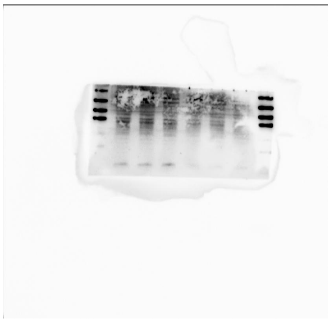

O:Bcl2

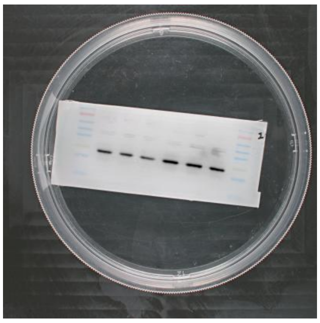

O:β-actin

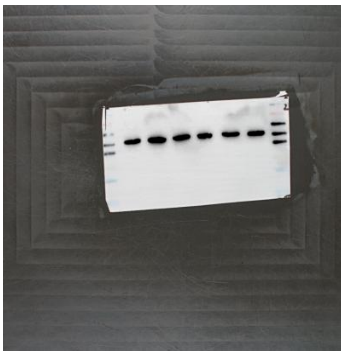

O:Bax

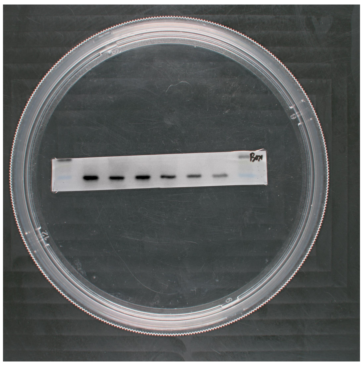

Fig5

I:chip

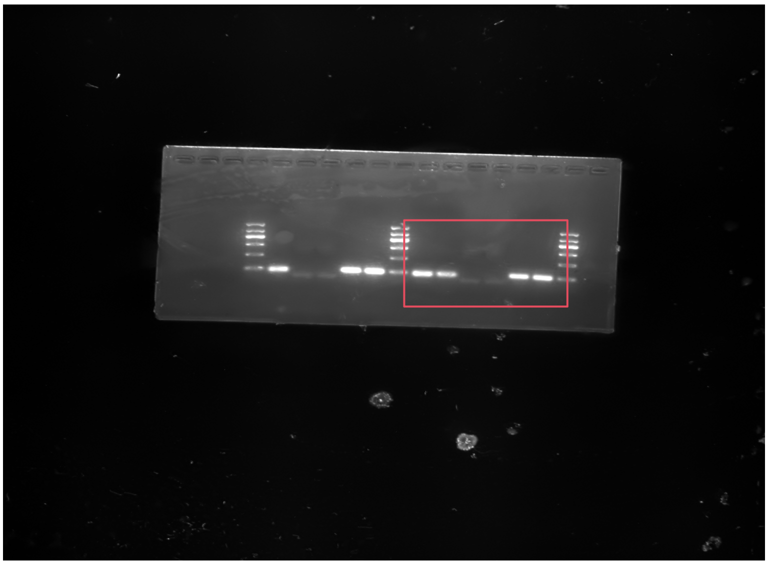

Fig6

E:P-stat3

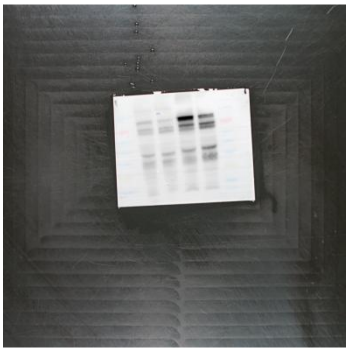

F:P-stat3

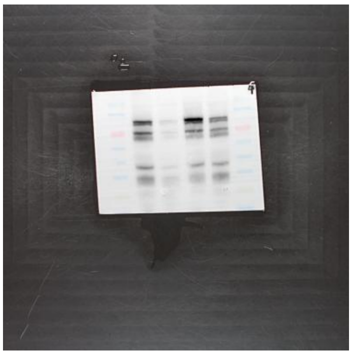

G:P-stat3

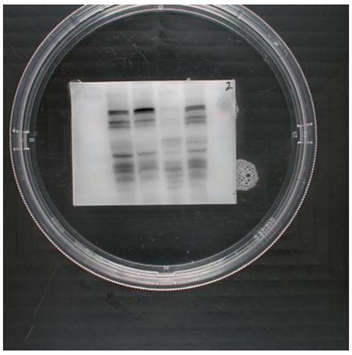

E:stat3

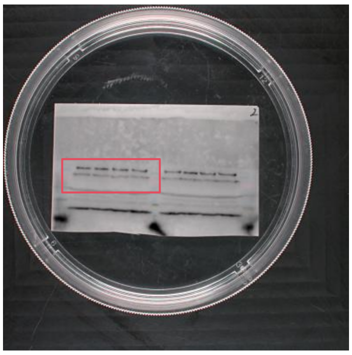

F:stat3

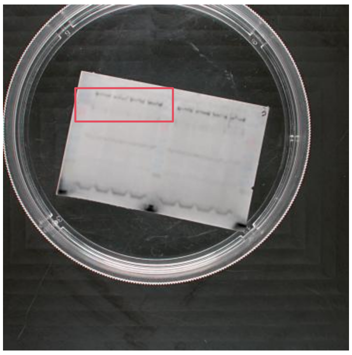

G:stat3

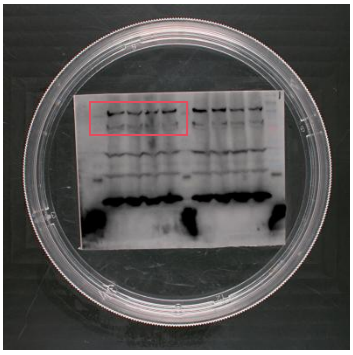

E:MAFF

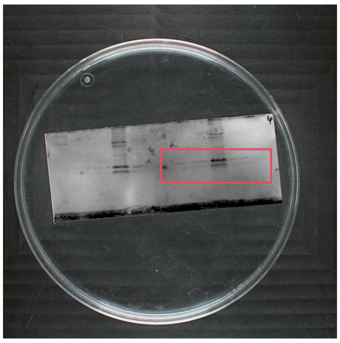

F:MAFF

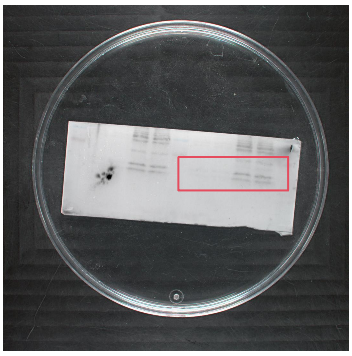

G:MAFF

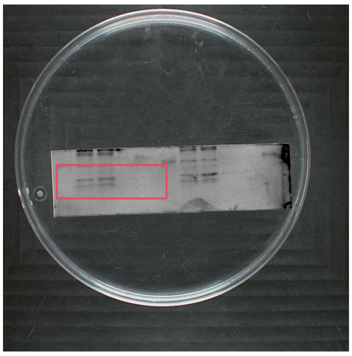

E:actin

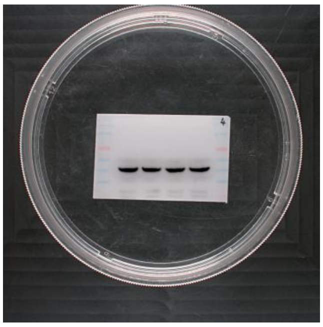

F:actin

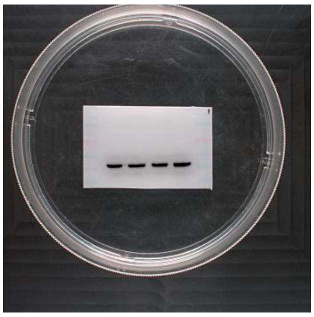

G:actin

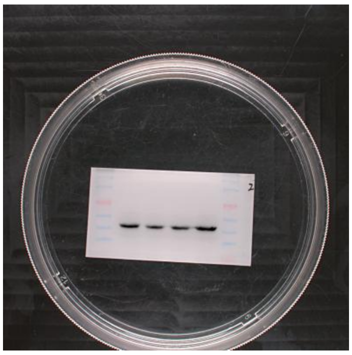

Fig7

G:MAFF

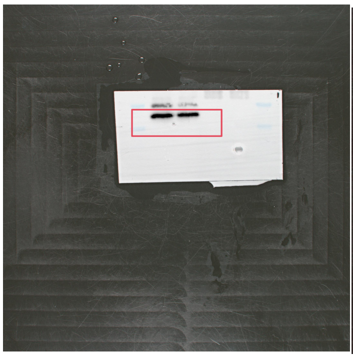

G:BACH1

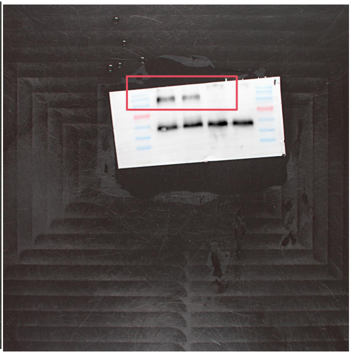

G:BACH1 MAFF

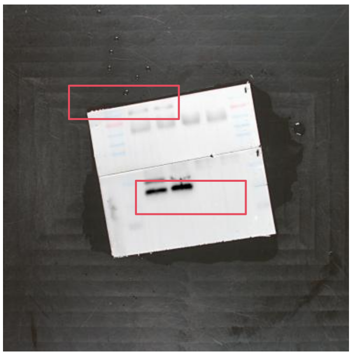

J:P-stat3

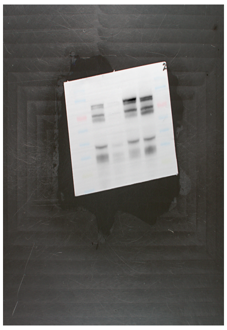

J:Stat3

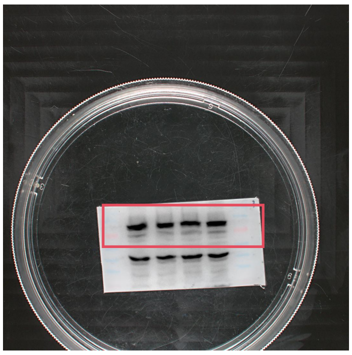

J:BACH1

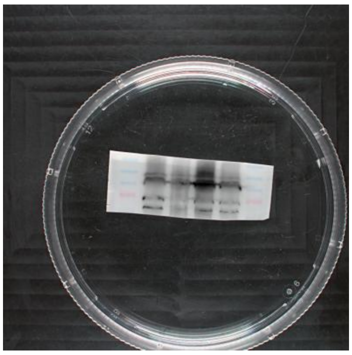

J:MAFF

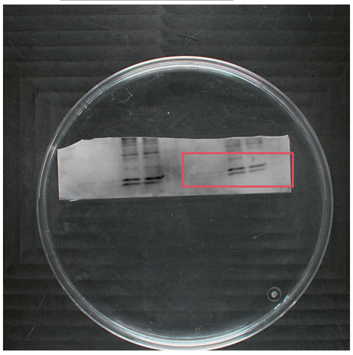

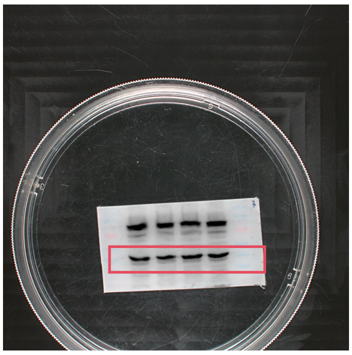

J:actin
