# Supplementary material for: MAFF alleviates hepatic ischemia–reperfusion injury by regulating the CLCF1/STAT3 signaling pathway
Source: Cell Mol Biol Lett. 2025 Apr 1;30:39. doi: 10.1186/s11658-025-00721-x (PMC11963299; doi:10.1186/s11658-025-00721-x)
Supplement: Supplementary file 2 — Additional File 2. [file 11658_2025_721_MOESM2_ESM.docx]

**Supplementary Data 1**

**RNA-sequencing**

Total RNA was extracted from the tissues using Trizol (Invitrogen, Carlsbad, CA,

USA) according to manual instruction. Subsequently, total RNA was qualifed and

quantifed using a Nano Drop and Agilent 2100 bioanalyzer (Termo Fisher Scientifc,

MA, USA). Te RNA library construction and subsequent RNA sequencing were

performed by BGI-Shenzhen, China. First-strand cDNA was generated using random

hexamer-primed reverse transcription, followed by a second-strand cDNA synthesis.

afterwards, A-Tailing Mix and RNA Index Adapters were added by incubating to end

repair. Te cDNA fragments obtained from previous step were amplifed by PCR, and

products were purifed by Ampure XP Beads, then dissolved in EB solution. Te

product was validated on the Agilent Technologies 2100 bioanalyzer for quality

control. Te double stranded PCR products from previous step were heated denatured

and circularized by the splint oligo sequence to get the fnal library. Te single strand

circle DNA (ssCir DNA) was formatted as the fnal library. Te fnal library was

amplifed with phi29 to make DNA nanoball (DNB) which had more than 300 copies

of one molecular, DNBs were loaded into the patterned nanoarray and single end 50

bases reads were generated on BGIseq500 platform (BGI-Shenzhen, China).The

sequencing data was fltered with SOAPnuke (v1.5.2) by and the clean reads were

mapped to the reference genome using HISAT2 (v2.0.4). Bowtie2 (v2.2.5) was

applied to align the clean reads to the reference coding gene set, then expression level

of gene was calculated by RSEM (v1.2.12). The heatmap was drawn by pheatmap

(v1.0.8) according to the gene expression in diferent samples. Essentially, diferential

expression analysis was performed using the DESeq2(v1.4.5) with false discovery

rate (FDR)-adjusted *P-*value (q-value)≤0.05. To take insight to the change of

phenotype, GO (http:// www.geneontology.org/) and KEGG (https://www.kegg. jp/)

enrichment analysis of annotated diferent expressed gene was performed by Phyper

(https://en.wikipedia.org/ wiki/Hypergeometric_distribution) based on

Hypergeometric test. Te signifcant levels of terms and pathways were corrected byFDR-adjusted P-value (*q*-value), with a rigorous threshold (*q*≤0.05) by Bonferroni.

All analysis were performed on the Dr. Tom analysis system constructed by

BGI-Shenzhen, China.

**CUT&Tag**

**Sample collection and library sequencing**

CUT&Tag for cell samples and Library preparation

Take out the cryovial and shake it in 37 ° C water bath to quickly thaw it within

1-2min.Cells were centrifuged for 5min at 600×g at room temperature. Cell activity

was detected with LUNA-FLTM and counted. CUT&Tag assay was performed as

described previously(Kaya-Okur, Wu et al. 2019). Briefly, the cells are bound to

Concanavalin A-coated magnetic beads, and the cell membrane is permeabilized by

Digitonin. The enzyme pA-Tn5 Transposase precisely binds the DNA sequence near

the target protein under the antibody guidance and results in

factor-targetedtagmentation. DNA sequence is tagmented, with adapters added at the

same time at both ends, which can be enriched by PCR to form the sequencing-ready

libraries. After the PCR reaction, libraries were purified with the AMPure beads and

library quality was assessed on the Agilent Bioanalyzer 2100 system.

**Sequencing (Novogene Experimental Department)**

The clustering of the index-coded samples was performed on a cBot Cluster

Generation System using TruSeq PE Cluster Kit v3-cBot-HS (Illumina) according to

the manufacturer ’ s instructions. The library preparations were sequenced on Illumina

Novaseq platform at Novogene Science and Technology Co., Ltd (Beijing, China) and

150 bp paired-end reads were generated.

**Data Analysis Quality control**

Raw data (raw reads) of fastq format were firstly processed using fastp (version

0.20.0). In this step, clean data (clean reads) were obtained by removing reads

containing adapter, reads containing ploy-N and low-quality reads from raw data. At

the same time, Q20, Q30 and GC content of the clean data were calculated. All the

downstream analyses were based on the clean data. Reads mapping to the referencegenome Reference genome and gene annotation files were downloaded from genome

website directly. Index of the reference genome was built using BWA(v0.7.12) and

clean reads were aligned to the reference genome using BWA mem. These reads were

then filtered for high quality (MAPQ ≥ 13),we also removed reads that were not

properly paired and with PCR duplicates. Only uniquely mapped(MAPQ ≥ 13) and

de-duplicated reads were used for further analysis.

**Peak calling**

All peak calling was performed with MACS2 (version 2.1.0) using ‘macs2 -q 0.05 -f

AUTO --call-summits--nomodel --shift -100 --extsize 200 --keep-dup all’. By default,

peaks with q-value threshold of 0.05 was used for alldata sets.

**Motif analysis**

Peaks were adjusted to the same size (500 bp) centered peak summits and motif

discoveries of these loci sequences were performed using findMotifsGenome.pl

program in HOMER v4.11 software with ‘-len 8,10,12,14 -gc -size given

-homer2 -dumpFasta’.

**Peak annotation**

The position of peak summit around transcript start sites of genes can predict the

interaction sites between protein and gene. ChIPseeker (Yu et al., 2015) was used to

retrieve the nearest genes around the peak and annotate genomic region of the peak.

Peak-related genes can be confirmed by ChIPseeker, and then Gene Ontology

(GO) enrichment analysis was performed to identify the function enrichment results.

GO enrichment analysis was implemented by the GOseq R package, in which gene

length bias was corrected. GO terms with corrected P-value less than 0.05 were

considered significantly enriched by peak-related genes. KEGG is a database resource

for understanding high-level functions and utilities of the biological system, such as

the cell, the organism and the ecosystem, from molecular-level information,

especially large-scale molecular datasets generated by genome sequencing and

otherhigh-throughput experimental technologies (http://www.genome.jp/kegg/). We

used KOBAS software to test the statistical enrichment of peak related genes in

KEGG pathways.

**Different peak analysis**

Peaks of different groups were merged using‘bedtools merge’.

We caltulated the mean RPM of each group in the merge peak. Only peaks with fold

change of RPM more than 2 were considered as differential peaks. Genes associated

with different peaks were identified using ChIPseeker.

**Reference**

Kaya-Okur, H. S., S. J. Wu, C. A. Codomo, E. S. Pledger, T. D. Bryson, J. G.

Henikoff, K. Ahmad and S. Henikoff(2019). "CUT&Tag for efficient epigenomic

profiling of small samples and single cells." Nat Commun 10(1): 1930.
