# Supplementary figures and images for: MAFF alleviates hepatic ischemia–reperfusion injury by regulating the CLCF1/STAT3 signaling pathway
Source: Cell Mol Biol Lett. 2025 Apr 1;30:39. doi: 10.1186/s11658-025-00721-x (PMC11963299; doi:10.1186/s11658-025-00721-x)

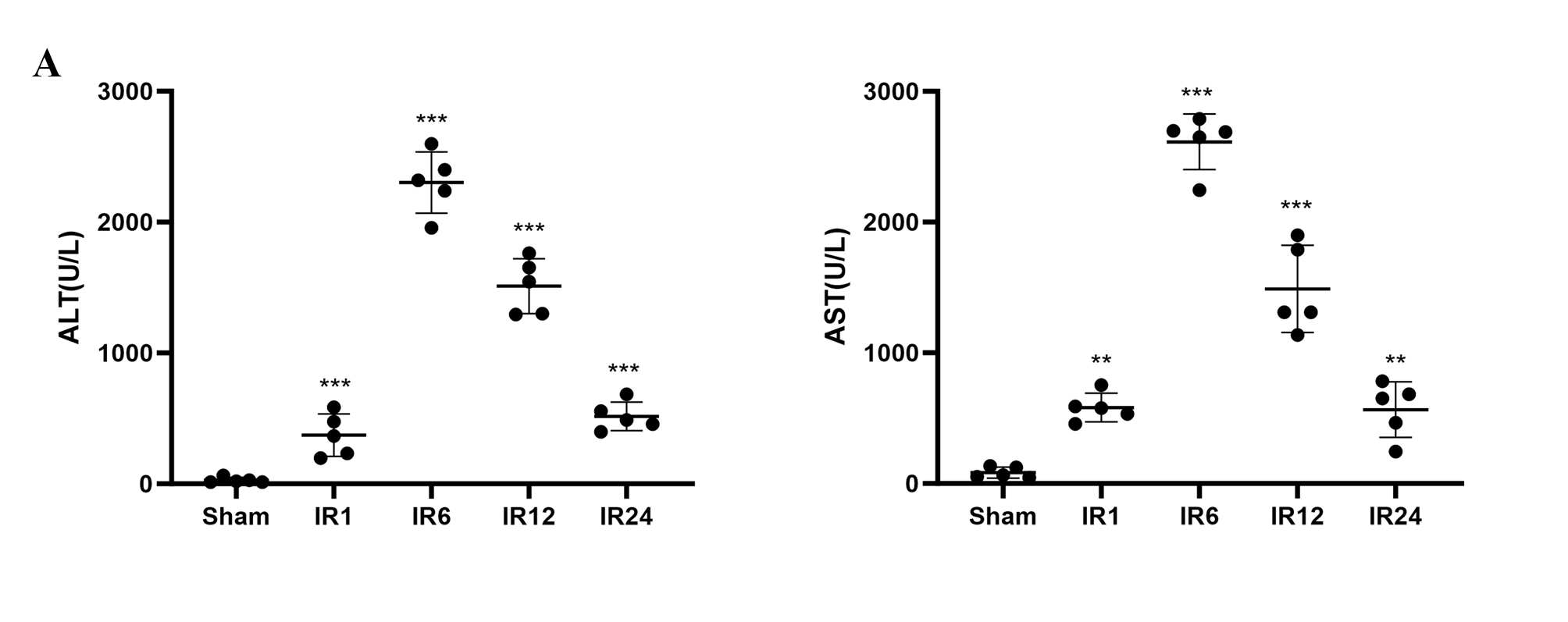

Supplement: Supplementary file 3 — Additional File 3. [file 11658_2025_721_MOESM3_ESM.tif]

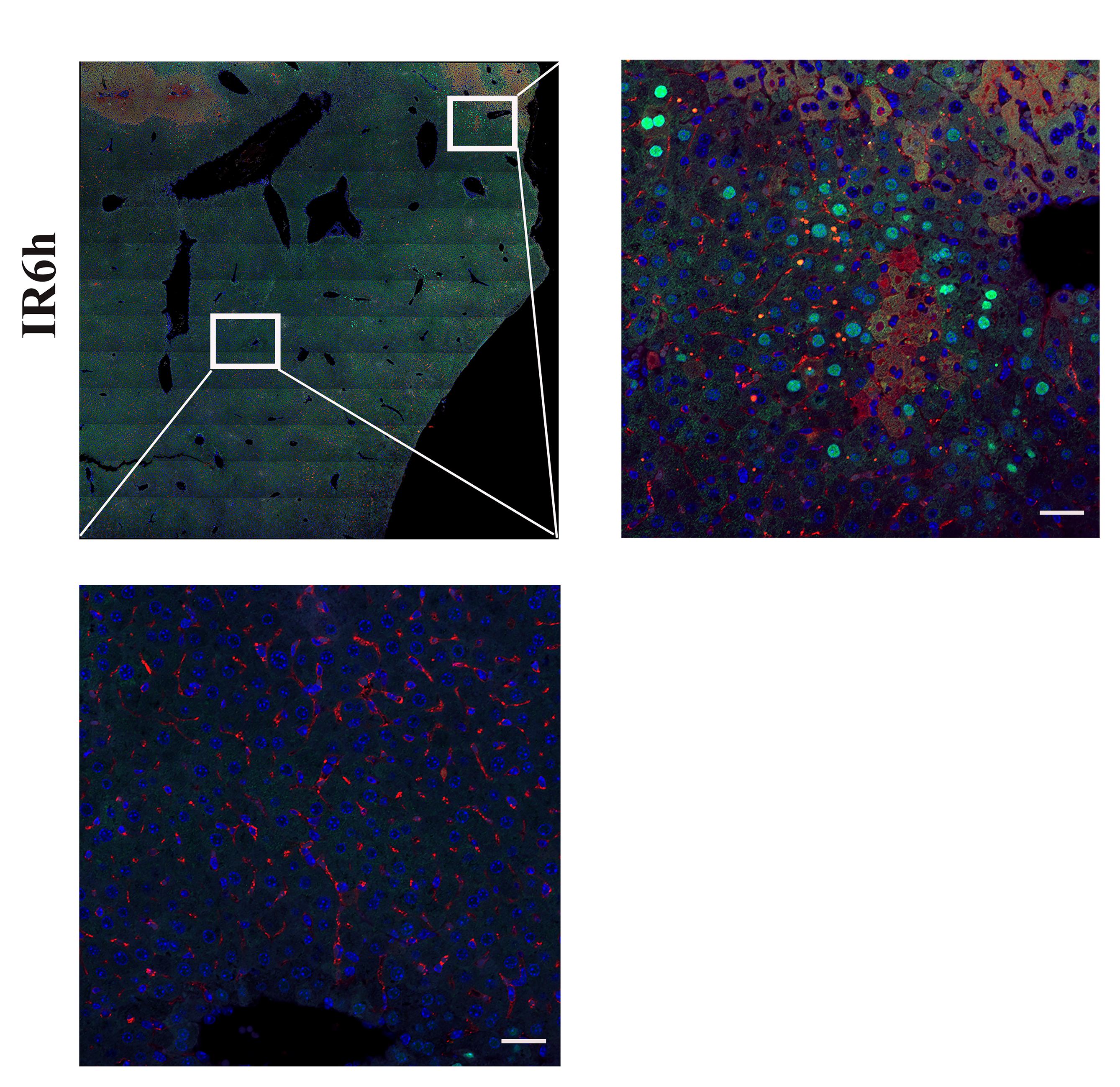

Supplement: Supplementary file 4 — Additional File 4. [file 11658_2025_721_MOESM4_ESM.tif]
