# Supplementary material for: MAFF alleviates hepatic ischemia–reperfusion injury by regulating the CLCF1/STAT3 signaling pathway
Source: Cell Mol Biol Lett. 2025 Apr 1;30:39. doi: 10.1186/s11658-025-00721-x (PMC11963299; doi:10.1186/s11658-025-00721-x)
Supplement: Supplementary file 5 — Additional File 5. [file 11658_2025_721_MOESM5_ESM.docx]

**Supplementary Figure 1. ALT and AST levels.**

**Supplementary Figure 2. Immunofluorescence was used to determine MAFF expression levels in different places.**
